# An integrated multiscale imaging workflow to resolve intracellular co-pathology in human FFPE brain tissue

**DOI:** 10.17879/freeneuropathology-2026-9581

**Published:** 2026-06-16

**Authors:** Sophie Schreiner, Mónica Miranda de la Maza, Morgane Darricau, David S. Bouvier

**Affiliations:** 1 National Center of Pathology, Laboratoire national de santé, Dudelange, Luxembourg; 2 Department of Infection and Immunity (DII), Luxembourg Institute of Health (LIH), Belval, Luxembourg; 3 Luxembourg Centre for Systems Biomedicine (LCSB), University of Luxembourg, Belval, Luxembourg

**Keywords:** FFPE brain tissue, Multiscale resolution, Immunohistochemistry, Multiplex immunofluorescence, High-content imaging, Neurodegeneration

## Abstract

Multiscale and multimodal analysis of *post-mortem* human brain tissue is essential for improving the characterisation of neurodegenerative diseases (NDDs). Brain bank repositories provide extensive collections of formalin-fixed paraffin-embedded (FFPE) tissue enabling investigation of cellular and subcellular alterations across NDDs. Although conventional and multiplex chromogenic immunohistochemistry (cIHC) support large-scale neuropathological assessment, they do not fully capture the spatial and cellular complexity. Immunofluorescence (IF) combined with confocal microscopy increases spatial resolution, but standard section thickness limits three-dimensional (3D) analysis.

Here, we present a sequential, multimodal and semi-automated workflow within a unified pipeline that enhances the multiscale definition of the neuropathological signature. Using neuronal and pathological markers as technical references, we demonstrate stepwise additive spatial information across modalities: from anatomical distribution using single cIHC, to two-dimensional combinatorial detection using multiplex cIHC, and to volumetric intracellular localisation using multiplex IF on thick sections.

When applied to human FFPE brain tissue, this workflow enables reproducible and scalable multiscale protein mapping. It supports consistent detection and spatial separation of multiple markers and links chromogenic pathology with high-resolution 3D fluorescence imaging, providing a practical framework for the integrated spatial analysis of neurodegenerative pathology and beyond.

## Introduction

Neurodegenerative disease (NDD) research relies on histological methods that preserve spatial context while resolving molecular heterogeneity across tissue, cellular, and subcellular scales [1]. Formalin-fixed paraffin-embedded (FFPE) human brain tissue is the most widely used preservation method in tissue preparation and brain banking and remains the gold standard material for neuropathological assessment [4,5]. Chromogenic immunohistochemistry (cIHC) is widely used in both diagnostic and research settings due to its robustness, low cost, and compatibility with standard brightfield microscopy [6,7]. However, conventional single-marker cIHC provides limited insight into the spatial and molecular context of protein expression [6]. Multiplex chromogenic and fluorescence-based imaging approaches extend single-marker analysis by enabling co-detection of multiple markers; however, in FFPE tissue, they remain constrained by inherent compromise in multiplexing capability and three-dimensional (3D) spatial resolution imposed by standard tissue thickness [8,9].

To overcome these complementary limitations, we developed a sequential, multimodal and semi-automated workflow that integrates single cIHC, multiplex cIHC, and 3D multiplex immunofluorescence (IF) within a unified experimental pipeline, enabling additive multiscale spatial information across modalities. The workflow is implemented on the semi-automated Ventana Discovery Ultra IHC/in situ hybridization (ISH) research platform (Roche Ventana Medical Systems, Tucson, AZ, USA) and combines standardized sectioning strategies with iterative imaging approaches, enabling scalable analysis from tissue-level mapping to subcellular resolution. We demonstrate how each step contributes distinct but complementary spatial information by using a set of representative cellular and pathological markers on NDD samples as technical examples. This information ranges from anatomical distribution to two-dimensional (2D) co-occurrence and, finally, 3D intracellular localisation.

We applied this workflow to human FFPE brain tissue from Alzheimer’s disease (AD) and Parkinson’s disease dementia (PDD), the most common NDDs with distinct but partially overlapping protein aggregation profiles [10]. Hippocampal and cortex samples of these cases were selected to provide robust and widely recognized pathological examples for evaluating the performance of multimodal and multiscale imaging in FFPE tissue.

We focused on established markers of neuronal integrity (MAP2, NeuN), microglial marker (Iba1), tau pathology (AT8), synuclein pathology (pSyn81A), amyloid pathology (4G8), granulovacuolar degeneration-associated changes (pSMAD2), and necroptosis-related signalling (pRIPK3) to test the ability of the workflow and resolve structurally diverse intracellular signatures across complementary staining modalities [11]. These marker sets were chosen to span different pathological protein aggregation, cellular compartments and cell types, thereby enabling systematic evaluation of detection, performance, and resolution across all workflow steps.

## Material & Methods

### 1. Human brain samples

This study was performed on human *post-mortem* FFPE hippocampal tissue obtained from the Douglas-Bell Canada Brain Bank (Douglas Mental Health University Institute, Montreal, QC, Canada) and the Netherlands Brain Bank (NBB, Amsterdam, The Netherlands). The use of this tissue for research was approved by the Douglas-Bell Canada Brain Bank, Netherlands Brain Bank ethics committee and by the University of Luxembourg (ERP 16–037 and 21–009). The FFPE hippocampal tissue from two AD (#1, #2) and one PDD (#3) case, along with FFPE prefrontal cortex (PFC) tissue from one AD patient (#4), were included in this study (**Table 1**). Neuropathological assessment was carried out by neuropathologists associated with the brain banks following established diagnostic frameworks, including ABC and Braak Lewy body (LB) staging [18,19].

**Table 1:** Case information and neuropathological data of included samples

Case	Pathological diagnosis	Sex	Age at death	Post-mortem delay (PMD/hours)	ABC Score/ BRAAK LB	Brain bank
#1	AD	M	87	10.8	A2B3C2	Montreal
#2	AD	F	86	3.8	A3B3C3	Netherland
#3	PDD	M	72	4	A0B1C0/ LB6	Netherland
#4	AD	F	96	26.5	A2B3C2	Montreal

Sample information and corresponding neuropathological data were obtained from brain banks.

### 2. Sectioning and slide preparation

To assess reproducibility and optimize methodological performance, serial sections of the FFPE tissue blocks were cut using a standard microtome and allocated to the different experimental conditions. The number of consecutive sections is defined by the planned staining panel and marker combinations. A subset of these sections was allocated to single and multiplex cIHC and cut at 5 μm. For multiplex IF, consecutive sections of varying thickness (5, 10, and 15 μm) were used to assess performance across section thicknesses, and additional 15 μm sections were prepared for duplex and triplex staining with high-resolution imaging. All sections were mounted on hydrophilic adhesion glass slides (Matsunami TOMO®, Cat# TOM-1190) and incubated at 60 °C for 1 hour prior to downstream experimental procedures.

### 3. Single chromogenic immunohistochemistry (single cIHC)

All experiments were performed using Ventana Discovery Ultra automated IHC/ ISH research platform (Roche Ventana Medical Systems, Tucson, AZ, USA). Reagents were loaded and used in the system according to the manufacturer’s recommendations, with washing steps performed between incubations using Reaction Buffer (Cat# 950–300).

To optimize antigen detection and establish appropriate primary antibody dilutions, single cIHC was performed using horseradish peroxidase (HRP)-based 3,3'-Diaminobenzidine (DAB) visualisation. Each antibody was optimized individually, and final working concentrations are provided in **Table 2**. All slides were counterstained with haematoxylin II (Cat# 790–2208) and bluing reagent (Cat# 760–2039) for 4 min each. Consecutively, all slides were washed, dehydrated through a series of alcohols and coverslipped using the Tissue-Tek Prisma® Plus and Tissue-Tek Film® (Sakura Finetek) automated coverslipping systems.

**Table 2:** Antibodies used for single and multiplex cIHC and multiplex IF

Antibodies	Source	Identifier	Dilution
Primary antibodies	Primary antibodies	Primary antibodies	Primary antibodies
Mouse monoclonal anti-NeuN, clone A60	Millipore	Cat# MAB377, RRID: AB_2298772	1:200
Rabbit polyclonal anti-MAP2	Millipore	Cat# AB5622, RRID: AB_91939	1:250
Rabbit polyclonal anti-Iba1, C-term	Wako	Cat# 019–19741 RRID: AB_839504	1:1000
Mouse monoclonal anti-phospho-Tau (Ser202, Thr205), clone AT8	Thermo Fisher Scientific	Cat# MN1020, RRID: AB_223647	1:1000
Mouse monoclonal anti-phospho-α-Synuclein (Ser129), clone 81A	Millipore	Cat# MABN826, RRID: AB_2904158	1:500
Mouse monoclonal anti-β-Amyloid, clone 4G8	BioLegend	Cat# 800712 RRID: AB_2734548	1:2000
Rabbit monoclonal anti-phospho-Smad2 (Ser465/467), clone A5S	Sigma-Aldrich	Cat# ZRB04953, RRID: N/A	1:200
Rabbit monoclonal anti-phospho-RIPK3 (ser227), clone D6W2T	Cell Signaling Technology	Cat# 93654, RRID: AB_2800206	1:200 (single cIHC) 1:100 (multiplex cIHC, IF)
Secondary antibodies	Secondary antibodies	Secondary antibodies	Secondary antibodies
DISCOVERY OmniMap anti-Rb HRP	Roche	Cat# 760-4311, RRID: AB_2811043	Ready to use*
DISCOVERY OmniMap anti-Ms HRP	Roche	Cat# 760-4310, RRID: AB_2885182	Ready to use*
DISCOVERY UltraMap anti-Rb Alk Phos	Roche	Cat# 760-4314, RRID: N/A	Ready to use*
DISCOVERY UltraMap anti-Ms Alk Phos	Roche	Cat# 760-4312, RRID: N/A	Ready to use*
DISCOVERY CM DAB kit	Roche	Cat# 760-159, RRID: N/A	Ready to use*
DISCOVERY Purple Kit	Roche	Cat# 760-229, RRID: N/A	Ready to use*
DISCOVERY Teal HRP kit	Roche	Cat# 760-247, RRID: N/A	Ready to use*
DISCOVERY Yellow Kit	Roche	Cat# 760-239, RRID: N/A	Ready to use*
DISCOVERY Rhodamine 6G Kit	Roche	Cat# 760-233, RRID: N/A	Ready to use*
DISCOVERY Cy5 Kit	Roche	Cat# 760-238, RRID: N/A	Ready to use*
DISCOVERY FAM Kit	Roche	Cat# 760-243, RRID: N/A	Ready to use*
DISCOVERY QD DAPI (RUO)	Roche	Cat # 760-4196, RRID: N/A	Ready to use*

Antibodies used for marker optimization, including source, catalog number (Cat#), research resource identifiers (RRID) and optimized primary antibody dilution, are listed. Staining was performed on the Ventana Discovery Ultra platform. *Vials ready-to-use purchased from Roche Ventana Medical Systems.

For single-plex DAB cIHC, sections were automatically deparaffinized and rehydrated on the instrument at 69 °C using EZ Prep solution (Cat# 950-102) in three consecutive 8 min cycles. Heat-induced epitope retrieval (HIER) was subsequently performed at 95 °C for 40 min using Cell Conditioning 1 buffer (CC1, Cat# 950-224). To suppress endogenous peroxidase activity and reduce nonspecific background staining, tissue sections were incubated with ChromoMap (CM) inhibitor from the Discovery CM DAB kit (Cat# 760-159) for 8 min at 37 °C.

Primary antibodies against neuronal nuclei (NeuN), microtubule-associated protein 2 (MAP2), ionized calcium-binding adapter molecule 1 (Iba1), phosphorylated tau (AT8), phosphorylated synuclein (pSyn81A), β-Amyloid (4G8), pSMAD2, and pRIPK3 were diluted in EnVision™ FLEX antibody diluent (Cat# K8006) and applied manually (**Table 2**), followed by a 60 min incubation. Signal detection was achieved using the appropriate OmniMap secondary reagents (anti-Mouse, Cat# 760-4310; anti-Rabbit, Cat# 760-4311) for 16 min and chromogenic development was achieved using the Discovery CM DAB kit (Cat# 760-159). The DAB reaction was developed sequentially using H₂O₂ CM (4 min), DAB CM (8 min), and Copper CM (4 min), yielding stable, high-contrast staining suitable for downstream analysis.

### 4. Multiplex chromogenic immunohistochemistry (multiplex cIHC)

Multiplex cIHC was performed to enable sequential chromogenic detection of multiple targets within the same tissue section using the Ventana Discovery Ultra platform (Roche Diagnostics), as explained before. All the multiplex protocols (two- and three-plex) followed the same initial steps of deparaffinization, HIER with CC1, and blocking with inhibitor CM as the single-plex.

In the two-plex stainings of pSMAD2/AT8, pRIPK3/AT8 and pSyn81A/AT8, after deparaffinization and HIER steps, one drop of Discovery inhibitor (Cat# 760–4840) was applied for 8 min. Sections were then incubated with anti-pSMAD2, anti-pRIPK3 or pSyn81A for 60 min, detected using OmniMap anti-rabbit HRP or OmniMap anti-mouse HRP for 16 min and developed using the Discovery Purple Kit (Cat# 760–229) with Discovery Purple (4 min) and H₂O₂ Purple (32 min). Prior to each sequential staining cycle within each panel, heat-mediated antibody denaturation was performed (100 °C, 24 min) using Cell Conditioning 2 (CC2; Cat# 950–223) to disrupt the primary antibody–HRP complex and minimize carryover, thereby preventing binding of subsequent chromophores to residual HRP activity. Sections were subsequently incubated with AT8 for 60 min, which was detected using OmniMap anti-mouse HRP for 16 min and developed using Discovery Green HRP Kit (Cat# 760-271) with a 4-min incubation with Green HRP Substrate, 16 min with Green HRP H2O2, and 32 min with Green HRP Activator. For pRIPK3/pSMAD2 two-plex, anti-pRIPK3 was stained with purple chromogen, as explained previously. Then, anti-pSMAD2 was applied and detected with Discovery Teal HRP chromophore (Cat# 760–247) using Teal HRP Substrate (4 min), Teal HRP H₂O₂ (32 min), and Teal HRP Activator (16 min). The last two-plex of pSMAD2/AT8 was performed following the same procedure but developing pSMAD2 with Discovery CM DAB kit and AT8 in Discovery Teal HRP chromophore.

The three-plex pSMAD2/AT8/MAP2 and pSMAD2/AT8/NeuN were performed first by applying pSMAD2 as explained above and visualised using the Discovery CM DAB kit. In a second step, AT8 was applied for 60 min, detected using OmniMap anti-mouse HRP for 16 min and developed using Discovery Purple Kit. Finally, anti-MAP2 was applied for 60 min followed by 16 min incubation with OmniMap anti-rabbit HRP and developed using Discovery Teal HRP, anti-NeuN was applied for 60 min followed by 12 min incubation with Discovery UltraMap anti-Ms Alk Phos (AP) (Cat# 760-4312) and chromogenic development was performed using the Discovery Yellow kit (RUO, Cat# 760–239) consisting of Yellow buffer (4 min) and Disco Yellow (44 min). The AT8/pRIPK3/pSMAD2 three-plex started with the incubation of AT8 for 60 min, as explained before, and its visualisation with Discovery Purple Kit. Then, pRIPK3 was applied for 60 min, followed by 16 min incubation with OmniMap anti-rabbit HRP and developed using Discovery Teal HRP. Finally, anti-pSMAD2 was incubated for 60 min, detected with 12 min incubation with Discovery UltraMap anti-Rb Alk Phos (AP) (Cat# 760-4314) and chromogenic development was performed using the Discovery Yellow kit. The Iba1/AT8/4G8 three-plex was initiated by applying anti-Iba1 for 60 min, followed by detection with OmniMap anti-rabbit HRP for 16 min and chromogenic development using the Discovery CM DAB kit. In a second step, AT8 was applied for 60 min, detected using OmniMap anti-mouse HRP for 16 min and developed using Discovery Purple Kit. Finally, 4G8 was applied for 60 min, detected using OmniMap anti-mouse HRP for 16 min and developed using Discovery Teal HRP.

Upon completion of the multiplex staining procedure, slides were counterstained with haematoxylin, dehydrated through graded ethanol and xylene, and coverslipped following the same protocol used for single-target IHC using the automated slide coverslipper Tissue-Tek Prisma® Plus and Tissue-Tek Film® (Sakura Finetek).

### 5. Multiplex immunohistofluorescence (multiplex IF)

Multiplex IF was implemented on the same platform. After deparaffinization, tissue sections underwent HIER at 95 °C for 64 min in CC1. Endogenous peroxidase activity and nonspecific background were suppressed by treatment with the Discovery inhibitor for 8 min at 37 °C.

Primary antibodies targeting Iba1, 4G8, AT8, MAP2, pSMAD2, and pRIPK3 were diluted in EnVision™ FLEX antibody diluent (Table 1) and applied manually for 60 min. Detection was performed using species-appropriate HRP-conjugated OmniMap secondary reagents (anti-Mouse; anti-Rabbit) for 12 min, followed by tyramide signal amplification with tyramide-conjugated fluorophores applied for 8 min. Fluorophores included Rhodamine 6G (R6G, Cat# 760-233, λ_ex ≈ 542 nm / λ_em ≈ 568 nm), Cyanine 5 (Cy5, Cat# 760-238, λ_ex ≈ 650 nm / λ_em ≈ 670 nm) and fluorescein amidite (FAM, Cat# 760-243, λ_ex ≈ 490 nm / λ_em ≈ 520 nm). Between fluorophore applications, a heat-mediated deactivation (HD, 100 °C, 24 min) with CC2 step was performed to minimize cross-reactivity and ensure signal specificity.

Three duplex stainings of increasing section thickness were performed for AT8 and Iba1, with fluorescence detection using Cy5 and FAM. In addition, duplex staining for AT8 and MAP2 was conducted as described above. To assess reproducibility across experimental conditions, three sections from an independent case were stained for AT8 and Iba1 with variation in antibody order (AT8/Iba1 vs. Iba1/AT8) and fluorophore combinations (Cy5/FAM and R6G/Cy5). Furthermore, a section from a third case was subjected to duplex staining for AT8 and pSyn81A using Cy5 and FAM.

The pSMAD2/AT8/MAP2 three-plex was performed first by applying pSMAD2 and developed with R6G, second AT8 developed with Cy5 and third MAP2 developed with FAM. A second case was processed using a pSMAD2/AT8/NeuN panel with identical fluorophore assignment (R6G/Cy5/FAM). Another pSMAD2 /AT8/pRIPK3 three-plex was carried out and developed using R6G, Cy5, and FAM, respectively. Additionally, an Iba1/AT8/4G8 three-plex was performed with Iba1 developed in Rhodamine, AT8 in Cy5, and 4G8 in FAM.

After completion of fluorescent labelling, slides were rinsed in dH₂O, air-dried at room temperature protected from light for 10 min, and coverslipped manually with ProLong™ Gold Antifade Mountant (Cat# P36934; Thermo Fisher Scientific) to preserve fluorescence.

### 6. Image acquisition and processing

Chromogenically stained FFPE tissue sections were imaged using brightfield microscopy on a Leica DM2000 LED microscope (Leica Microsystems) equipped with 5×, 10×, 20×, and 40× objectives and an LMS Leica Flexcam i5 camera.

Fluorescently labelled sections were imaged in three dimensions using confocal microscopy. Z-stack acquisitions were obtained with Zeiss LSM800 confocal microscopes using 20× air (Plan-APOCHROMAT 20×/0.8) and 40× oil (EC Plan-NEOFLUAR 40×/1.3 Oil DIC) objectives. All 3D image stacks were visualised and analysed in Imaris software (version 11.0.1) after performing channel-specific intensity normalization and background subtraction to remove noise, ensuring accurate representation of signal and spatial relationships within the tissue.

## Results

### Workflow part 1 – Tissue-level mapping of multiple pathological markers using serial single chromogenic staining

**Figure 1: Distribution of dendritic, tau-related, granulovacuolar and necroptosis-associated markers in hippocampal neurons:**
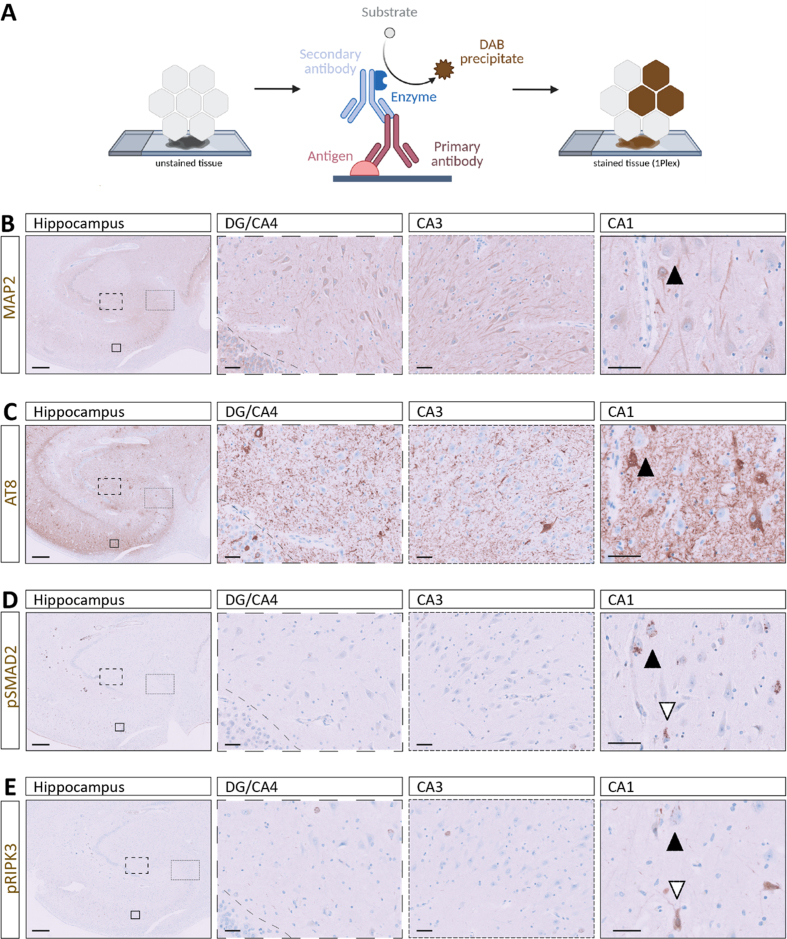
**A**. Schematic overview of the staining workflow from unstained tissue through DAB chromogen development to the final stained section. **B–E**. Representative single cIHC stainings across the hippocampus, including DG/CA4, CA3, and CA1 subfields: **B**. MAP2, **C**. AT8, **D**. pSMAD2, and **E**. pRIPK3. Black and white arrows indicate the same neuron across stainings in serial sections, enabling comparative assessment of distinct pathological features within the same anatomical context. All stainings were performed using case #1. Scale bars (left to right): 500 μm and 50 μm.

To establish the first level for our multiscale analysis, we applied single cIHC to serial FFPE hippocampal sections using an established neuropathological procedure (**Fig. 1A**). This step establishes the reliability of the markers and characterizes their topographic neuropathological pattern at both regional and subregional level. To challenge the efficiency of our multimodal approach, we selected tau pathology and granulovacuolar degeneration (GVD) as representative neuronal pathologies but characterised by distinct subcellular aggregation patterns. Serial sections (5 μm each) derived from the same tissue block, the hippocampus of an AD case (A2B3C2, male, 87-year-old, PMD: 10.8 h), were prepared and stained individually for MAP2 (neuronal morphology), AT8 (PHF-tau, pSer202/pThr205), pSMAD2 (granular inclusions), and pRIPK3 (necroptosis marker) (**Fig. 1B–E**).

Immunolabelling patterns were evaluated across DG/CA4, CA3, and CA1 subfields to enable subregional comparison of marker distribution (**Fig. 1B–E**). Single cIHC allowed robust visualisation of individual markers, with MAP2 delineating neuronal architecture, while AT8, pSMAD2, and pRIPK3 each displayed distinct and heterogeneous pathology-associated staining patterns within neuronal populations of the hippocampal formation. These immunolabelling enabled identification of distinct marker-positive neuronal populations and facilitated mapping of their distribution across hippocampal subregions (**Fig. 1**, black and white arrows). Comparable staining patterns for NeuN (neuronal nuclei), AT8, and pSMAD2 were observed in another AD hippocampal sample (A3B3C3, female, 86-year-old, PMD: 3.8 h), supporting the reproducibility of the approach (**Suppl. Fig. 1A**). Additionally, we used PFC tissue of another AD patient (A2B3C2, female, 96-year-old, PMD: 26.5 h) to exhibit both intracellular tau pathology, stained by AT8, and extracellular amyloid deposits, visualized by 4G8, and the local microglia stained by the canonical marker Iba1 (**Fig. 4A**). The need of this approach across disease contexts was further demonstrated in a PDD case (A0B1C0, male, 72-year-old, PMD: 4 h), which showed synuclein pathology, stained by pSyn81A, as well as tau pathology, stained by AT8 (**Fig. 4D**).

As illustrated in the AD CA1 region, comparative evaluation of serial sections suggested co-occurrence of multiple pathological markers within the same anatomical territory and potentially within the same neuronal populations (**Fig. 1B–E**). However, as these markers were assessed on consecutive sections, this approach relies on indirect alignment, precluding definitive assessment of intracellular co-localisation or subcellular spatial relationships.

### Workflow part 2 – Multiplex chromogenic staining enables concurrent detection of multiple markers within a single FFPE section

**Figure 2: Multiplex cIHC reveals intracellular co-localisation of cellular and functional markers in CA1 neurons:**
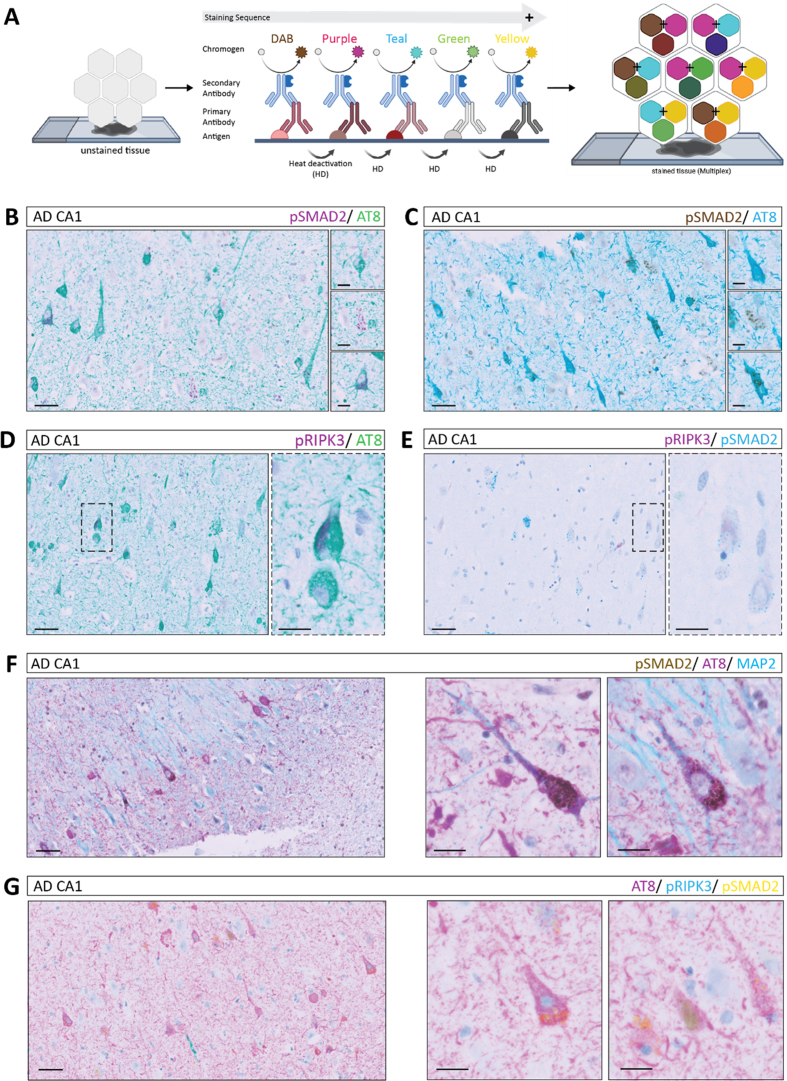
**A**. Schematic overview of the multiplex cIHC staining workflow from unstained to stained tissue, indicating the sequential order of chromogen application and heat-mediated deactivation (HD) between staining cycles, as well as the colour combinations and potential merged colours. **B, C**. Representative co-staining of pSMAD2 and AT8 using different chromogen combinations. **D**. Co-staining of pRIPK3 and AT8. **E**. Co-staining of pRIPK3 and pSMAD2. **F**. Triplex staining of pSMAD2, AT8 and MAP2. **G**. Co-staining of AT8, pRIPK3 and pSMAD2. All staining were performed using case #1. Scale bars (left to right): 50 μm and 20 μm.

To overcome its limitations and extend the workflow toward direct multi-marker assessment, we used multiplex cIHC to simultaneously visualise multiple markers, each labelled with a distinct chromogen, within the same hippocampal tissue section (**Fig. 2A**). A critical parameter of this approach is the selection and combination of chromogens, as the signal appearance depends on the translucency, relative target abundance and antibody concentration. Information obtained from single-marker DAB staining was used to guide both chromogen selection and antibody concentration for each marker, ensuring optimal contrast and intensity in the multiplex assay. Strong, opaque colours such as DAB, green or purple produce more intense, well-defined signals, whereas more translucent colours such as teal or yellow produce lighter staining that may overlap with other chromogens. In addition, the sequential order of antibody application, determined by the manufacturer, influenced the final signal composition and enabled controlled adjustment of marker representation (**Fig. 2A**).

To exemplify this step of the workflow, co-staining of pSMAD2 (purple) and AT8 (green) in a consecutive hippocampal section of the same AD case enabled discrimination of distinct marker-positive cell populations as well as visualisation of overlapping signals within the same cell (**Fig. 2B**). These signals appeared as mixed colours resulting from chromogen overlap. Therefore, it was indicative of apparent co-distribution rather than definitive intracellular co-localisation. These observations were consistent across different colour combinations, including DAB with teal (**Fig. 2C**), demonstrating the robustness of multiplex cIHC while underscoring the importance of chromogen selection for reliable interpretation. Similar observations were obtained for the combination of pRIPK3 (purple) and AT8 (green). Here, multiplex cIHC enabled detection of both individual and overlapping staining patterns within cell populations. However, resolving low-abundance signals such as pRIPK3, in contrast to a broader AT8 staining, remained challenging (**Fig. 2D**). To further assess the capability of multiplex cIHC to distinguish markers with partially similar staining patterns, duplex staining of pRIPK3 (purple) and pSMAD2 (teal) was performed. While both markers could be visualised within the same cells, differences in staining intensity and pattern, particularly for low-abundance targets, limited precise discrimination of intracellular localisation (**Fig. 2E**), illustrating a technical constraint of chromogenic multiplexing. In addition, duplex staining of pSyn81A (purple) and AT8 (green) in a PDD sample further confirmed the applicability of the workflow across different disease contexts and intracellular marker combinations (**Fig. 4E**).

Extension to triple staining further showed the performance of multiplex cIHC to combine structural and pathological markers. Simultaneous visualisation of pSMAD2 (DAB), AT8 (purple) and MAP2 (teal) enabled identification of neuronal populations exhibiting multiple pathological marker signals (**Fig. 2F**). In this colour scheme, overlapping chromogens produced mixed colour tones, such as dark blue from purple and teal, providing visual cues for potential co-distribution at the cellular level. However, interpretation remained dependent on colour contrast and signal intensity. Similarly, three-plex staining of AT8 (purple), pRIPK3 (teal) and pSMAD2 (yellow) allowed large-scale evaluation of multiple pathological features within the same tissue section. Rare triple-positive cells and limited colour discriminability, particularly in densely stained cytoplasmic regions, restricted reliable identification of all markers within individual cells (**Fig. 2G**). Comparable multiplex staining patterns were observed in an independent AD case using a three-plex combination of pSMAD2 (DAB), AT8 (teal) and NeuN (yellow) stained by different chromogens, and a triplex staining of another AD case combining Iba1-labeled microglia (DAB), intracellular tau (purple) and extracellular amyloid plaques (teal) in the PFC (**Fig. 4B**), supporting the reproducibility of the approach.

Overall, this workflow step represents a major advance in the neuropathological characterisation by enabling the direct and two-dimensional visualisation of multiple pathologies within individual cells. Nevertheless, interpretation remains constrained by chromogen overlap, limited colour separation and the inability to resolve fine intracellular structures or provide volumetric information. These challenges motivated the integration of an additional complementary step within the workflow, capable of providing higher-resolution and three-dimensional characterisation of neuropathological markers.

### Workflow part 3 – Multiplex IF on 15 μm thick FFPE sections combined with confocal microscopy enables high-resolution and volumetric analysis of intracellular marker distribution

**Figure 3: Semi-automated multiplex IF and 3D imaging of intracellular co-pathologies:**
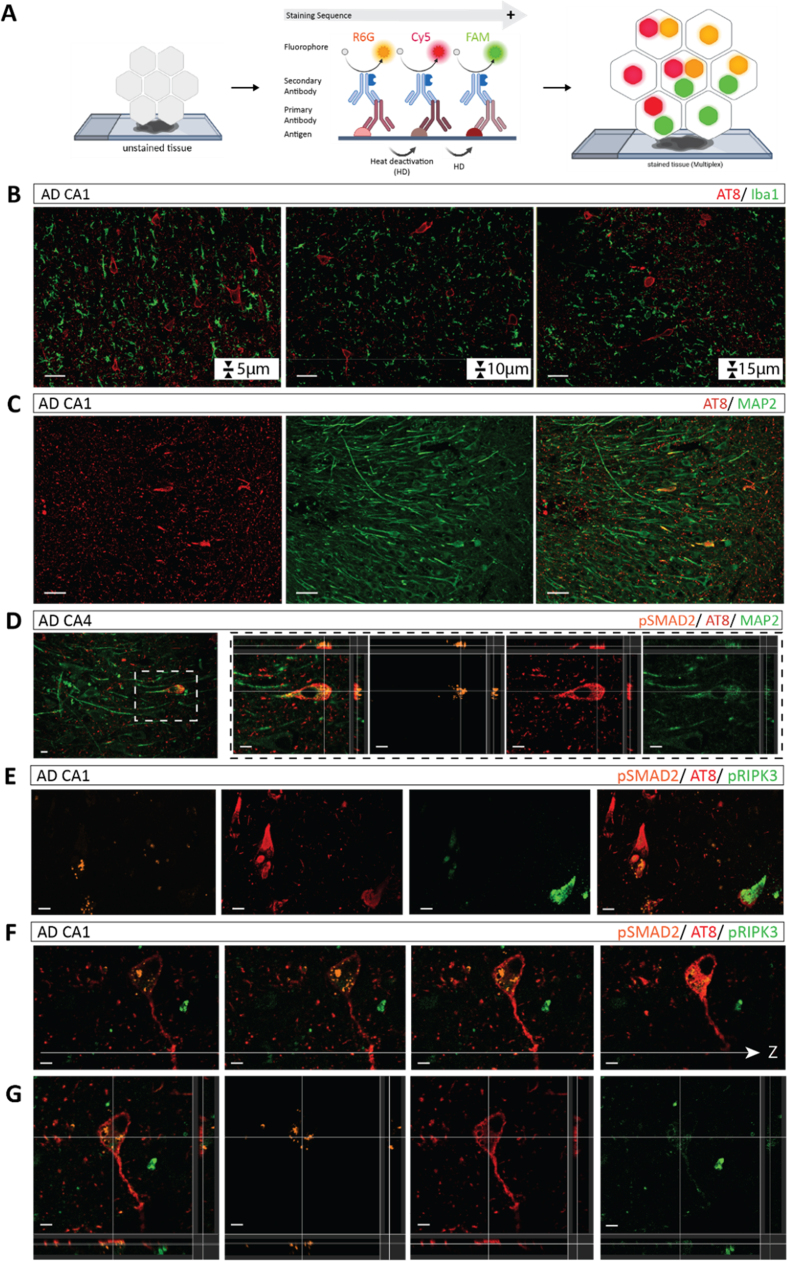
**A**. Schematic overview of the automated multiplex IF staining workflow. In the stained tissue, individual signals are visualised in separate fluorescence channels (FAM: 488 nm - green, R6G: 555 nm - orange, and Cy5: 647 nm - red). **B**. Serial sections of different tissue thicknesses stained for AT8 and Iba1. **C**. Representative double IF staining of AT8 and MAP2 in CA1 neurons. **D**. Representative multiplex IF staining of pSMAD2, AT8 and MAP2 in a CA1 neuron with 3D visualisation. **E**. Triplex staining of pSMAD2, AT8 and pRIPK3 demonstrating intracellular co-occurrence of tau pathology, GVD–related signalling, and necroptosis-associated marker. **F**. Illustrating the suitability of the approach, imaging across section z-depth in one multiplex IF triplex staining of pSMAD2, AT8 and pRIPK3 and the 3D reconstruction. All stainings were performed using case #1. Scale bars: 50 μm (B, C), 15 μm (E) and 10 μm (D, F, G).

To complete the final step of our workflow, we applied semi-automated multiplex IF on the FFPE hippocampal tissue (**Fig. 3A**). As an initial optimisation step, we systematically assessed the effect of section thickness on the staining quality and signal homogeneity. We compared consecutive sections of 5 μm, 10 μm and 15 μm thickness from the same FFPE block labelled in duplex for AT8 and Iba1 as a representative example. Using identical confocal imaging settings for z-stack acquisitions, antibody labelling patterns and signal intensities were comparable across all thicknesses in terms of qualitative distribution and overall signal detectability. The 15 μm section provided an optimal balance between 3D spatial information and signal intensity. This thickness was therefore selected as the standard for subsequent IF experiments (**Fig. 3B**). The robustness of duplex staining across experimental variations, including independent tissue samples, antibody order and fluorophore selection, was further confirmed (**Suppl. Fig. 1C**).

We next applied multiplex IF to duplex labelling of AT8 (Cy5) and MAP2 (FAM) to assess signal separation and marker discrimination. The use of spectral distinct fluorophores combined with confocal detection enabled clear separation of channels and reliable identification of both markers within the same tissue section (**Fig. 3C**). Selection of appropriate secondary antibodies was guided by the spectral detection capabilities of the confocal microscope, which restrict the range of fluorophores that can be efficiently excited and detected. In our workflow, we employed secondary antibodies conjugated to the fluorophores FAM (488 nm), R6G (555 nm), and Cy5 (647 nm) to match to the available confocal detection channels and minimize spectral overlap. The multiplexing capability could be further expanded within the limits of spectral separation and detector configuration, as the system is programmable for up to six targets [36].

**Figure 4: Workflow exemplified across co-existing pathological markers in AD and PDD cases:**
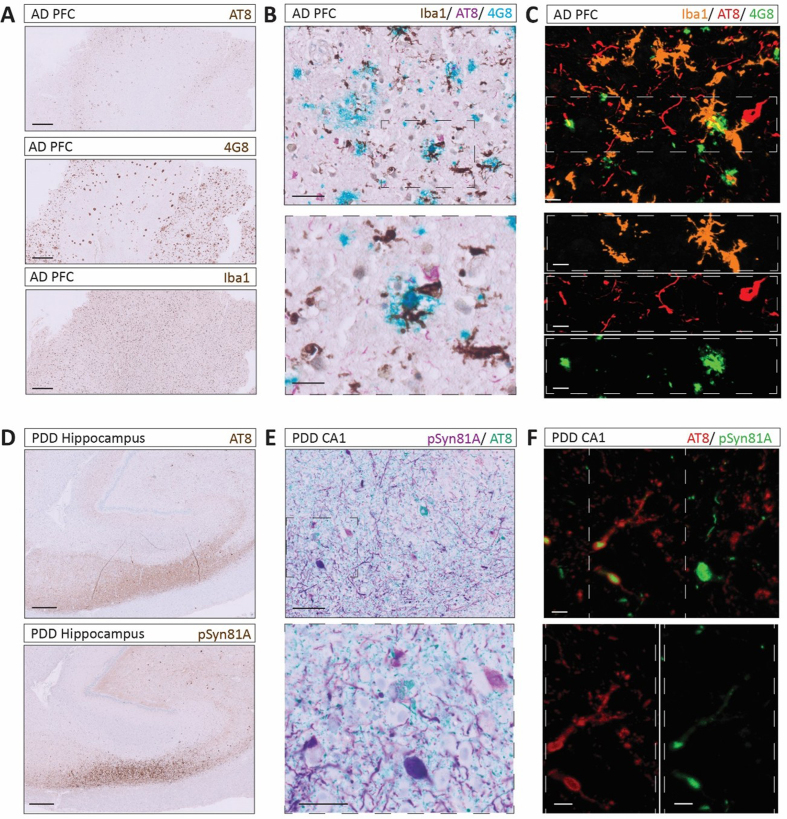
**A**. Single cIHC of AT8, 4G8 and Iba1 in the PFC of an AD case. Scale bar: 500 μm; case #4. **B**. Triplex cIHC staining for Iba1, AT8 and 4G8. Scale bar (up to down): 50 μm and 20 μm; case #4. **C**. IF triplex staining for Iba1, AT8 and 4G8. Scale bar: 10 μm; case #4. **D**. Single cIHC of AT8 and pSyn81A in the hippocampus of a PDD case. Scale bar: 500 μm; case #3. **E**. Multiplex cIHC duplex staining for pSyn81A/AT8. Scale bar (up to down): 100 μm and 50 μm; case #3. **F**. IF duplex staining for AT8 and pSyn81A. Scale bar: 10 μm; case #3.

Triplex staining was subsequently incorporated, making full use of our 3D confocal imaging at 20× and 40× magnification allowing investigation of subcellular co-pathologies (**Fig. 3D,E**). The multiplex IF staining of pSMAD2 (R6G) and AT8 (Cy5) in combination with MAP2 (FAM) revealed consistent staining patterns compared to multiplex cIHC allowing to assess the subcellular distribution of pSMAD2 vesicles and AT8 tangles within neurons of which boundaries were stained by MAP2. Triplex combination such as pSMAD2 (R6G), AT8 (Cy5), and pRIPK3 (FAM) enabled the simultaneous visualisation of multiple intracellular markers within individual neurons across optical planes, highlighting their specific subcellular distribution (**Fig. 3E–G**). In contrast to chromogenic approaches, fluorescence-based detection allowed clear separation of signals, enabling 3D discrimination of markers within distinct subcellular compartments, illustrating the improved capacity of multiplex IF to distinguish closely associated intracellular structures. The reproducibility and broader application of the workflow across disease contexts, brain region and marker combination were further demonstrated by validation in an independent AD case (pSMAD2 (R6G) / AT8 (Cy5) / Iba1 (FAM); **Suppl. Fig. 1B**), in a PDD case (AT8 (Cy5) / pSyn81A (FAM); **Fig. 4F**) and in the PFC tissue of an additional AD case (Iba1 (R6G) / AT8 (Cy5) / 4G8 (FAM); **Fig. 4C**).

## Discussion

**Figure 5: A unified pipeline of a semi-automated, sequential and multimodal staining workflow to enhance neuropathological investigations:**
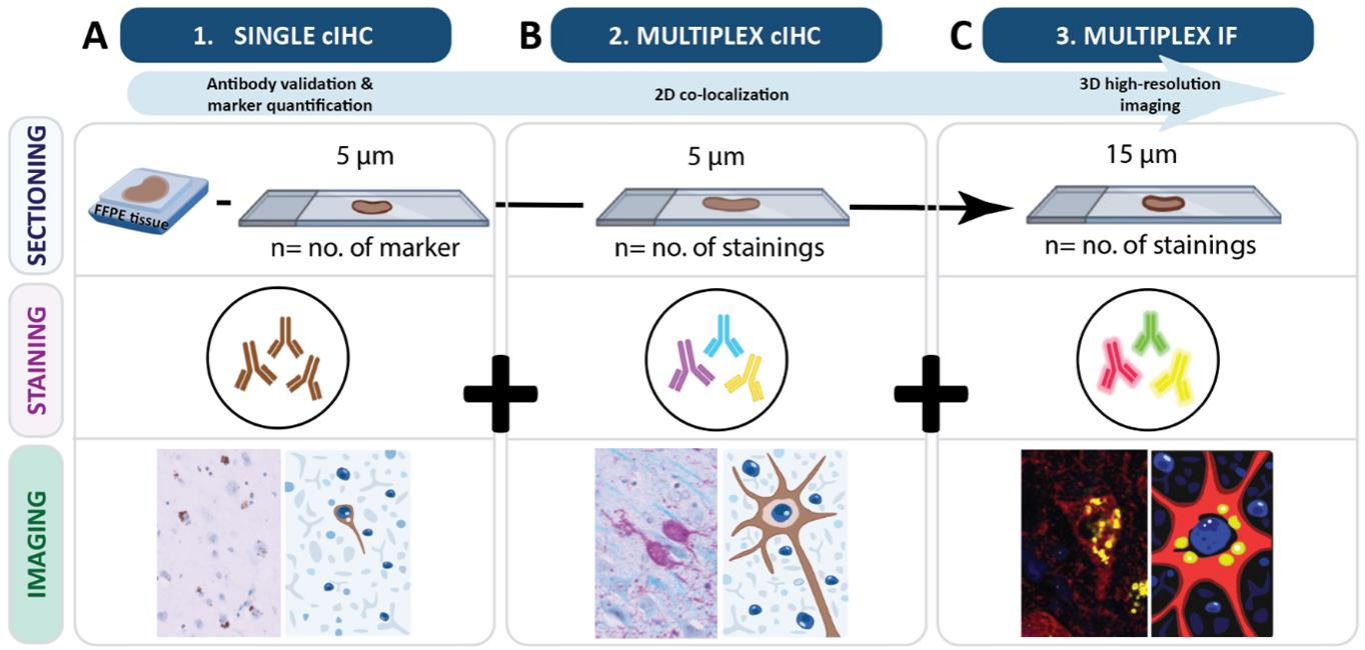
Schematic illustrating the integrated workflow combining single cIHC, multiplex cIHC, and multiplex IF with 3D confocal imaging to enable multiscale visualisation across tissue depths. All sections were derived from the same FFPE tissue block. **A. **In workflow Step 1, single cIHC was performed on 5 μm sections, with the number of sections depending on the number of markers. Single DAB stain provides large-scale imaging of tissue architecture used for antibody validation and marker quantification. **B.** Step 2, multiplex cIHC, was also performed on 5 μm sections, with the number of sections determined by the planned multiplex combinations. This approach enables cellular resolution to analyse co-localisation of intracellular proteins. **C.** Step 3, multiplex IF, was performed on 15 μm sections, with the number of sections adjusted according to the planned multiplex fluorescence stainings. Multiplex IF stainings can be used in 3D high-resolution imaging, which facilitates detailed mapping of co-localised pathological markers.

In this methodological study, we established a sequential, multimodal and multiscale workflow to improve the analysis of co-pathologies in FFPE human brain samples. By integrating single cIHC, multiplex cIHC and multiplex IF within a unified experimental pipeline, this approach enables neuropathological investigation across complementary levels of resolution (**Fig. 5**). By systematically comparing chromogenic (single and multiplex) and high-resolution fluorescence-based imaging modalities, we show that their combined application enhances the resolution of complex intracellular co-pathologies from anatomical region or sub-region to 3D subcellular organisation (commented in **Table 3**). Collectively, these findings highlight the value of integrating complementary imaging strategies to achieve a more comprehensive and multiscale understanding of neuropathological processes in human brain tissue. While our observations demonstrate technical feasibility, the analysis presented here is primarily qualitative, and further studies are required to validate the biological relevance of these observations in a larger collection of samples.

**Table 3:** Comparison of histopathological methods for the detection of protein pathologies in human FFPE tissue

Method	Main characteristic	Advantage	Limitation
Single chromogenic immunohistochemistry (single cIHC)	Single antigen detection using chromogenic substrates (e.g. DAB)	+ Robust and well-standardized + Preserves tissue morphology + High specificity for target proteins + Compatible with FFPE human tissue + Cost-effective + Requires commonly available equipment	– Limited to a single marker per section – Sequential staining may not accurately capture true co-localisation – Insufficient spatial resolution (e.g., GVBs) – Challenges with signal amplification – Increased tissue requirement
Multiplex chromogenic immunohistochemistry (multiplex cIHC)	Sequential application of multiple chromogens on the same section	+ Detection of multiple markers in situ + Preserves tissue architecture + Compatible with FFPE human tissue + Longer lasting signal (stains more resistant than fluorophores)	– Limited number of usable chromogens – Colour overlap complicates interpretation, especially within the same cell (difficult target co-localisation) – 2D visualisation due to thin sections
Multiplex immunohisto-fluorescence (multiplex IF)	Labelling of multiple targets using fluorophore-conjugated secondary antibodies	+ Higher spatial resolution than chromogenic IHC + Multiplexing capability + Enhanced sensitivity + 3D visualisation	– Autofluorescence and epitope masking in FFPE brain tissue – Photobleaching – Often manual and low throughput – Higher cost and specialized expertise

### All for one: unified pipeline to enhance co-pathology characterisation in FFPE brain tissue

Single cIHC is a standard method for diagnostic, prognostic, predictive, and therapeutic assessments, including tumour classification, evaluation of inflammation, and neuropathological analysis. In neuropathology, it is routinely applied in both diagnostic and research settings [1,20] and is compatible with established *post-mortem* neuropathological report and staging systems such as Braak staging, Thal phase, and the ABC score [18,28]. Its main strengths include robustness, low cost, compatibility with standard brightfield microscopy (**Table 3**), and straightforward implementation in clinical laboratories [7]. Consistent with these advantages, single cIHC enabled robust detection of individual pathological markers and support large-scale mapping of protein distribution. It is also well suited for quantitative measurements of anatomical patterns of marker expression with digital pathology approaches. However, this approach does not permit direct analysis of intracellular co-distribution or colocalisation (**Table 3**).

Multiplex cIHC partially overcomes this limitation by enabling simultaneous visualisation of a limited number of markers within a single section [7,22,31], improving co-detection compared with sequential single cIHC and supporting analyses when tissue is limited (**Table 3**). However, its interpretability remains constrained by chromogen overlap, limited multiplexing capacity, and the 2D nature of staining, which restricts resolution of precise intracellular and volumetric relationships.

Our experimental approach shows that automated multiplex IF addresses several of these constraints and enables reproducible volumetric visualisation of multiple pathological markers within the same neuron. Increased section thickness facilitates 3D visualisation of small intracellular compartments or organelles, such as GVD-like structures, within the boundaries of individual cells. While sectioning thick (10–15 μm) FFPE human brain tissue presents technical challenges, including a higher risk of tissue folding, tearing, or partial breakage, these issues were mitigated by optimizing tissue temperature, intermittently cooling the tissue block, and performing cuts at reduced speed. Alternating thick sections with standard 5 μm sections further maintained section stability and quality. Importantly, even sections up to 15 μm did not exhibit detectable reductions in signal intensity for any evaluated markers, indicating efficient antibody penetration and preserved antigenicity. Its application to FFPE human brain tissue is often limited by autofluorescence, antibody-dependent variability, and workflow complexity [32]. Here, tissue autofluorescence was negligible, and individual fluorescent channels remained clearly separable. Despite these advantages, several technical considerations remain. Repeated staining cycles may not be compatible with all antibodies, and epitope stability should be validated for each application. In addition, thick-section imaging increases acquisition time and generates large datasets, which may limit throughput without optimized imaging and semi-automated analysis pipelines.

Building on these capabilities, our workflow combines serial sectioning, semi-automated staining cycles on one automated slide stainer, and complementary brightfield and 3D high-resolution confocal imaging to generate reproducible multiplex labelling while preserving tissue morphology, structural integrity, and antigenicity.

We show the applicability of this multimodal workflow using tau pathology and GVD as representative pathological features in the human hippocampus. These markers were consistently detected across all workflow steps, with single cIHC providing large-scale mapping of individual markers across hippocampal subregions. Multiplex cIHC enabled 2D analysis of co-distribution, allowing the identification of a subpopulation of neurons with concomitant pSMAD2, AT8, and pRIPK3 pathology, while multiplex IF on thicker FFPE sections resolved their distinct 3D subcellular localisation within individual neurons. Similar spatial resolution could be observed in the analysis of 4G8, AT8 and Iba1, showing the distribution of local pathological burden in the glial microenvironment. While this study focuses on a limited set of markers associated with tau pathology, amyloid pathology, synuclein pathology and GVD, the workflow is further applicable and could be extended to other proteins, cell-type markers, and signalling pathways.

### Semi-automated staining platforms: workflow flexibility and multiplexing capacity

Semi-automated staining platforms such as the Ventana Discovery Ultra are already established and applied in neuroscience [2,33]. Compared with manual immunostaining, the semi-automated protocol allows sequential staining of multiple antibodies, including those derived from the same host species, through repeated stripping and restaining cycles. Besides, automation reduces operator-dependent variability and maintains consistent staining intensity across sections and batches, which is particularly relevant for comparative studies. Several automated multiplex staining platforms are currently available including Dako Omnis Immunostainer (Agilent), BOND RX Automated Research Stainer (Leica), VALENT® Automated Staining Platform (Biocare), CNT330 Automated IHC Stainer (Celnovte), COMET™ (Lunaphore Technologies), and CellScape™ (Canopy Biosciences, Bruker). While systems such as the COMET™ or CellScape™ extend multiplexing capacity through cyclic or imaging-based approaches, they are primarily optimized for defined panel workflows with limited protocol adaptability. Platforms designed for high-throughput clinical or routine diagnostic use similarly offer less flexibility for iterative protocol optimization. The Ventana Discovery Ultra, by contrast, allows staining parameters to be individually adjusted for each slide, which is particularly advantageous for complex applications requiring customized experimental workflow. The resulting output is directly compatible with downstream spatial profiling technologies enabling the integration of protein-level data with spatial context [36,37]. Importantly, the pipeline is not restricted to a single platform and other automated staining systems can be incorporated at individual steps. For instance, cyclic multiplexing platforms such as CellScape™ could be integrated to further extend the number of markers.

## Conclusion

By integrating chromogenic IHC and multiplex IF, this sequential, multiscale workflow bridges tissue-level pathology with high-resolution intracellular organization, enabling reproducible and scalable analysis of co-pathologies in FFPE brain tissue, with applications in neurodegenerative disease and broader relevance to diverse brain conditions.

## Data availability

The data generated during the current study are available from the corresponding author upon reasonable request.

## Consent for publication

All authors have consented for the publication of manuscript.

## Author contributions

DSB conceived and supervised the study. SSc performed the experiments. SSc performed image acquisition and processing. SSc prepared the figures. SSc, MMM and DSB wrote the manuscript. SSc, MMM, MD and DSB contributed to the final version of the manuscript.

## Conflict of interest statement

The authors report no competing interests.

## Supplementary material

Supplementary Figure 01.  (PDF file, 675 KB).

## Supplementary Material

Supplementary Figure 01. (PDF file, 675 KB)
