# Supplementary material for: An integrated multiscale imaging workflow to resolve intracellular co-pathology in human FFPE brain tissue
Source: Free Neuropathol. 2026 Jun 16;7:13. doi: 10.17879/freeneuropathology-2026-9581 (PMC13276616; doi:10.17879/freeneuropathology-2026-9581)
Supplement: Supplementary file 1 [file freeneuropathol-07-13-9581-s1.pdf]

Supplementary material

Supplementary Figure 01

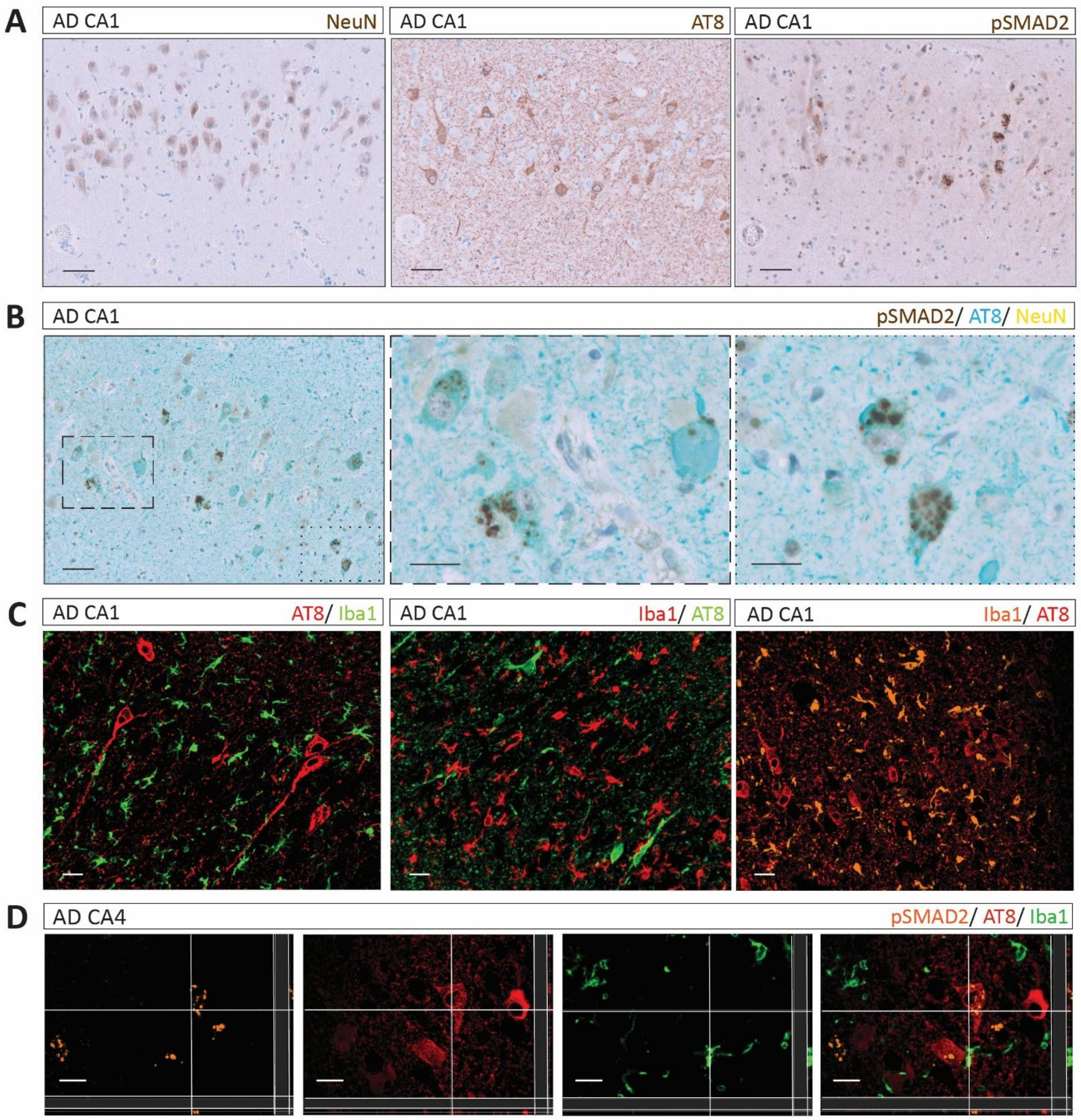

Suppl. Figure 1: Validation of workflow robustness across independent cases and marker combinations. A. Single cIHC of serial sections showing NeuN, AT8, and pSMAD2. Scale bar: 50 µm; case #2. **B**. Multiplex cIHC three-plex staining for pSMAD2, AT8, and NeuN. Scale bars: 50 µm (overview), 20 µm (inset); case #2. **C**. IF duplex staining of AT8 and Iba1 with different staining configurations (AT8/Iba1 and Iba1/AT8) using Cy5/FAM and R6G/Cy5 fluorophore combinations. Scale bar: 50 µm; case #2. **D**. IF three-plex staining for pSMAD2, AT8, and Iba1. Scale bar: 20 µm; case #2.

Copyright: © 2026 The author(s). This is an open access article distributed under the terms of the Creative Commons Attribution 4.0 International License (https://creativecommons.org/licenses/by/4.0/), which permits unrestricted use, distribution, and reproduction in any medium, provided the original author and source are credited, a link to the Creative Commons license is provided, and any changes are indicated. The Creative Commons Public Domain Dedication waiver (https://creativecommons.org/publicdomain/zero/1.0/) applies to the data made available in this article, unless otherwise stated.
